# Identifying SARS-CoV-2 Regional Introductions and Transmission Clusters in Real Time

**DOI:** 10.1093/ve/veac048

**Published:** 2022-06-16

**Authors:** Jakob McBroome, Jennifer Martin, Adriano de Bernardi Schneider, Yatish Turakhia, Russell Corbett-Detig

**Affiliations:** Biomolecular Engineering and Genomics Institute, University of California, Santa Cruz, USA; Biomolecular Engineering and Genomics Institute, University of California, Santa Cruz, USA; Biomolecular Engineering and Genomics Institute, University of California, Santa Cruz, USA; Electrical and Computer Engineering, University of California, San Diego, USA; Biomolecular Engineering and Genomics Institute, University of California, Santa Cruz, USA

**Keywords:** phylogeography, phylodynamics, genomic epidemiology, COVID-19, SARS-CoV-2, phylogenetic methods, Cluster-Tracker

## Abstract

The unprecedented SARS-CoV-2 global sequencing effort has suffered from an analytical bottleneck. Many existing methods for phylogenetic analysis are designed for sparse, static datasets and are too computationally expensive to apply to densely sampled, rapidly expanding datasets when results are needed immediately to inform public health action. For example, public health is often concerned with identifying clusters of closely related samples, but the sheer scale of the data prevents manual inspection and the current computational models are often too expensive in time and resources. Even when results are available, intuitive data exploration tools are of critical importance to effective public health interpretation and action. To help address this need, we present a phylogenetic heuristic which quickly and efficiently identifies newly introduced strains in a region, resulting clusters of infected individuals, and their putative geographic origins. We show that this approach performs well on simulated data and yields results largely congruent with more sophisticated Bayesian phylogeographic modeling approaches. We also introduce Cluster Tracker (https://clustertracker.gi.ucsc.edu/), a novel interactive web-based tool to facilitate effective and intuitive SARS-CoV-2 geographic data exploration and visualization across the United States. Cluster-Tracker is updated daily and automatically identifies and highlights groups of closely related SARS-CoV-2 infections resulting from transmission of the virus between two geographic areas by travelers, streamlining public health tracking of local viral diversity and emerging infection clusters. The site is open-source and designed to be easily configured to analyze any chosen regions, making it a useful resource globally. The combination of these open-source tools will empower detailed investigations of the geographic origins and spread of SARS-CoV-2 and other densely-sampled pathogens.

## Introduction

The massive scale of the SARS-CoV-2 sequencing effort has revealed deep inadequacies in our current methodology for phylogenetic analysis. Tools designed to work on small, sparse, static datasets have adapted poorly to the demands of a pandemic where tens of thousands of new genome sequences are generated and shared daily (Hodcroft et al 2021). Some have made progress by adopting generalized statistical methods built for large data such as random forest regression (O'Toole et al), while others have continued to improve on existing methods (Gill et al 2020, Vöhringer et al 2021), but phylogenetic solutions capable of scaling to millions of samples need to be developed. While our group, among others, has laid the groundwork for pandemic-scale phylogenetics (Dellicour et al 2021, Maio et al 2021, McBroome et al 2021, Schneider et al 2020, Shchur et al 2021, Turakhia et al 2021, Ye et al 2021) much remains to be done to translate evolutionary inferences to public health understanding and action.

The unprecedented scale of the genomic sequencing effort requires novel approaches to evolutionary, medical, and public health inference. Some groups have developed phylogenetically informed statistics for identifying mutations associated with increased transmissibility and other fitness-related parameters (Richard et al 2021, van Dorp et al 2020). In other cases, simple methods- such as the assaying of groups of identical samples- have been successfully applied to identify superspreader events and similar infection clusters (Bello et al 2022, Gómez-Carballa et al 2020). Unfortunately, many analyses still lack scalable or phylogenetically informed approaches.

The intersection of geography and phylogenetics, phylogeography, has often relied on heavily downsampled and static trees or limiting their analysis to early stages of the pandemic (Dellicour et al 2021, du Plessis et al 2021, Lemey et al 2020, Lemey et al 2021, Lemieux et al 2021, Ragonnet-Cronin et al 2021, Rito et al 2020). Some authors have analyzed several tens of thousands of samples with a divide and conquer approach, subdividing the overall tree by lineage and combining separately inferred results (McCrone et al 2021). Others have had similar success tracking the introduction and spread of a distinct new lineage over the first weeks after its emergence (Kraemer et al 2021). While useful for assessing transmissions between countries and major introductions, downsampling limits our ability to assign specific samples to regional infection clusters or identify clusters of potential interest. Even creative techniques taking advantage of phylogenetic tree structure to make analysis more tractable will not always be applicable and are limited in their ability to scale to millions of samples across dozens of regions. Additionally, much of these analyses are not readily interpretable for an efficient public health response, lacking intuitive visualization and data exploration tools. There is therefore a significant need for fast, automated, scalable and interpretable phylogeographic approaches for an effective public health response to emerging situations.

To address this need, we present here a phylogenetically-informed summary heuristic (the “regional index”), implementation (matUtils introduce), and data exploration and visualization tool (Cluster Tracker: https://clustertracker.gi.ucsc.edu/) for identifying introduction events and associated clusters of descendants in a given region. Our approach can be used to efficiently identify infection clusters and evaluate transmission dynamics across dozens of regions and millions of samples. Results obtained using this method are congruent with gold-standard Bayesian analyses and are accurate when applied to simulated data. Our visualization platform enables researchers and public health workers to explore new SARS-CoV-2 introductions across the USA, updated daily with all available global public data. This work will empower real time research and public-health applications of genomic epidemiology during the SARS-CoV-2 pandemic and beyond.

## Results and Discussion

### Cluster Concept and Definitions

A cluster, in terms of our analytical approach, is a set of closely-related samples from the same region and descended from a common ancestor with a regional introduction event. Under our definition, the complete set of actively circulating pathogens in a region will be composed of one or more genetically distinct clusters, which resulted from unique introduction events. In the phylogenetic tree, they appear as a set of leaves (samples) from a given geographic region that are descended from a shared common ancestor. A cluster may be monophyletic or paraphyletic, depending on whether some descendants of the cluster common ancestor left the geographic region. We consider location, or region, as a categorical state across the phylogenetic tree. A regional transmission event is where a child node is from a different region than the parent node. These patterns reflect cases of infected travelers moving between regions, followed by local transmission and eventual sampling of a number of descendant infections.

**A Heuristic for Identifying Introductions and Clusters**

The core of our heuristic is the "regional index", which is a weighted summary of the composition of descendants of a node of a phylogenetic tree. Intuitively, if all descendents of an internal node were found in region A, we would assume that the ancestor represented by that internal node was circulating in region A. Similarly, if we sampled a virus from region A which had exactly the inferred genome for this internal node, we would assume the ancestor represented by this node was in region A. The same logic would apply if no descendents were in region A. Therefore, by computing a heuristic which ranges from 0 to 1 based on the genetic distance to and composition of downstream descendents under a binary model of region membership, we can effectively approximate our intuition that the viral ancestor represented by that node was inside or outside a given region. It is defined as

$$Regional\ Index\ (C) = \frac{1}{1+\frac{\frac{Di}{Li}}{\frac{Do}{Lo}}}$$

where "$L_i$" is the number of downstream leaves that are in a given region, "$D_i$" is the minimum total branch length to a leaf descendent in the focal region, and "$L_o$" and "$D_o$" are the same for out-of-region leaves (Figure 1). On a tree inferred using maximum-parsimony, total branch length is equivalent to the distance in mutations between the query node and the descendant leaf.

We apply additional rules to handle cases where C is undefined or can't be computed. When a descendent leaf is genetically identical to the internal node and is in-region, C is 1. Similarly, when a genetically identical leaf is out-of-region, we treat C as 0. When such identical children exist both in and out of the region, we treat the node as in-region, as some infection with this genome must have existed in that region. We do not apply this index calculation to leaf nodes, which do not have children, and assume simply that the leaf is either in or out of the region as a given. This requires that each leaf included in the analysis be accompanied by accurate geographic location metadata.

This heuristic has several useful behaviors. For example, a sample identical to a specific internal node will always confer complete confidence about the location of that node, as we have sampled one genome that is identical to the ancestor directly. This can effectively identify nested clusters, where a new group of infections resulting from a regional introduction in turn produce clusters in other regions. It also accounts for the number of leaves downstream in our heuristic, on the assumption that introductions of a strain from one region to another require the lineage to be locally circulating in the origin region, but not necessarily lead to significant local transmission in the target region. This reduces the overall number of introductions we infer. If we account for the number of descendents, internal nodes will generally be assigned to the dominant region if distances are similar, reducing the number of consecutive reciprocal regional transmissions that might be inferred otherwise. Our heuristic strikes a balance between the principles of descendent composition and genetic distance, allowing us to efficiently analyze a large phylogenetic tree with minimal metadata.

Once indices for a given region have been calculated for each node, the second step is to identify clusters of samples putatively associated with an introduction. This is accomplished on a per-sample basis. The path from the sample to root is traversed and the indices for each ancestor being in the focal region is noted. Generally, the index declines from 1 to 0 along the ancestry path from leaf to root. The introduction point is called where the index for an ancestor being in-region is below 0.5, or the root, whichever is encountered first. 0.5 is our natural cutoff, representing the index value in a scenario where the composition and distance of downstream samples in and out of the region are equivalent, but can be adjusted by the user to modify cluster calling behavior. Once each sample has an ancestor chosen as the introduction node, they are grouped into clusters that share their ancestral introduction node. Generally, a larger threshold value will lead to more, smaller clusters, while a lower threshold value will lead to fewer, larger clusters.

As this heuristic is independent and specific to a region, it can be computed for an arbitrary number of regions across a single tree in parallel. When multiple regions are included, origins of putative clusters can be identified after introduction points are found by examining index scores across all other regions for the origin node and noting the region with the highest index. This metric can be calculated for one region of any size in a single post-order traversal with dynamic programming (see Methods), which makes it very fast to compute even on extremely large phylogenies with expansive regions.

**Evaluation of Our Heuristic Method**

Our implementation is part of the matUtils online phylogenetics package (McBroome et al 2021) and uses the efficient mutation annotated tree protocol buffer format and associated library (Turakhia et al 2021). To test runtime efficiency conditioned on a tree, we applied random subsampling and recorded time to compute our heuristic for a single region. We found that it takes less than forty five seconds on a single thread even for trees of more than two million samples (Supplementary Table 1).

To validate our results, we performed simulations consistent with viral evolutionary dynamics with inter-region dispersal events using the tools VGSim (Shchur et al 2021) and phastSim (Maio et al 2021) (see Methods). We found that our heuristic with default parameters recovered the true geographic location of internal nodes up to 99.8% of the time under realistic conditions for SARS-CoV-2 across an exactly correct bifurcating tree. We further attempted to model our ability to correctly recover clusters on a simulated tree with collapsed branches and realistic mutation rates for SARS-CoV-2. In comparing the clusters we recovered with the true set, we obtained an adjusted Rand index (Rand 1971) of up to 0.999. This suggests that our approach is generally quite accurate, though high migration rates or extremely low mutation rates can be confounding, as these scenarios are associated with minimal geographic and phylogenetic signal respectively (Supplementary Table 2; See Methods). More practically, this implies that our method will perform best when within-region transmission is substantially more common than between-region transmission (as in *e.g.,* country-level or state-level analyses).

To compare our results to widely used but much slower (days to months) analyses, we used our method to replicate a published phylogeographic analysis for the SARS-CoV-2

pandemic. Alpert et al used Bayesian phylogeography (Lemey et al 2009) to identify 23 distinct introductions of B.1.1.7 into the United States as of March 4th 2020. We obtained their subsampled tree and applied our heuristic using country labels to define the relevant regions (see Methods). With our method, we exactly replicated their identified clusters (Adjusted Rand Index 1.0). Alpert et al additionally predicted “sink” states, or the state to which each of the 23 introductions initially transmitted. We find that for all 23 clusters, samples in the identified sink state are closest or tied for closest in branch length to our inferred introduction point. This suggests that our approach can produce results congruent with more complex statistical models in a fraction of the time.

Another relevant method used in similar situations and that scales well to larger phylogenies is parsimony reconstruction, where region membership is treated as a character trait and inferred across the tree using the standard Fitch-Sankoff algorithm (Sankoff et al 1975, Vöhringer et al 2021, Volz et al 2021). This is more efficient than Bayesian approaches, but is heavily influenced by variation in sampling and low mutation rates relative to sampling and transmission. We performed a simple parsimony reconstruction based on the Fitch algorithm (Fitch 1977) similar to that of Volz et al on simulated data (Supplementary Table 3). We found that while parsimony performs as well or better than our heuristic on well resolved trees, when the average number of mutations per node is less than one and polytomies are common (as in SARS-CoV-2) our approach has greater accuracy. Our approach is more efficient than the Fitch algorithm because it requires only a single traversal of the phylogeny to compute.

**Global SARS-CoV-2 Transmission Dynamics and Infection Clusters**

Using our method, we traced transmission clusters in 102 countries from across the world (Figure 2A) using the global parsimony phylogenetic tree, built from 5,563,847 available sequences on GISAID (Shu et al 2017), GenBank (Sayers et al 201), and COG-UK (Lancet Microbe 2020) on 11-28-2021 (see Methods). Cluster size is highly skewed (Figure 2C), with approximately 20% of distinct regional clusters containing 89% of samples. This suggests that the majority of novel introductions do not lead to the establishment of a new locally-circulating strain, consistent with previous findings (du Plessis et al 2021).

Global contributions to sequence repositories are notably biased, with 51% of all samples belonging to either the USA or the United Kingdom (Figure 2B). This is a significant restriction on global transmission analysis, especially as the inference of the origin of a cluster is highly dependent on robust sequencing at the origin (see Methods). We therefore narrowed the next step of our analysis to the United States, which has robust and relatively comprehensive sequencing across each state as well as detailed state-level metadata for the vast majority of available samples.

**SARS-CoV-2 Transmission Into and Across the USA**

We identified more than three hundred thousand distinct state-level SAR-CoV-2 infection clusters in the United States over the course of the pandemic, as of November 2021 (Figure 3). Approximately 84% of these clusters have an assigned origin using our method (see Methods). Only 7% of our clusters appear to be of international origin, with the majority reflecting transmission within the USA. Mexico and Canada are among the most common international origin regions, in line with expectations given their long land borders (Supplementary Table 4).

England is also relatively common, likely because it is very well sampled. This indicates that it is possible that some clusters originate from less sampled intermediate regions and are assigned to the UK or other highly sampled locations. This suggests that relative sequencing effort in a given region is an important bias with respect to accurately identifying the origins of newly identified clusters and results should be interpreted with caution. International introductions rates are correlated with higher total sampling and therefore population size, particularly for California, Texas, New York, Massachusetts, and Florida (Figure 3B).

Within the USA, introductions come from a mix of neighboring states and high-population travel centers (Supplementary Table 5). We attempt to mitigate sampling biases- resulting from larger populations, higher case rates, increased sequencing, or other factors that are not specific to geography- by calculating a log-fold enrichment for rates of introduction from a given source region (see Methods; Figure 4). Note that while log-fold enrichment may reveal spatial relationships, it does not reflect the absolute importance of a region as a source or sink of viral transmission.

As with results from international introductions, we also find an enrichment for introductions that originate in geographically adjacent states. Log-fold enrichment is more than five times greater for neighboring states than for non-neighboring states within the USA (p=1.5e-117, Mann-Whitney U). Simple counts of inferred introductions are also enriched to a lesser extent between geographically adjacent states (p=2.2e-16, Mann-Whitney U). This suggests that SARS-CoV-2 transmission over interstate land borders is a major mechanism for spread within the USA. These results are largely in line with previous results in other viruses (Kozińska et al 2019) and SARS-CoV-2 (Tiwari et al 2021), suggesting that this heuristic is capturing and summarizing true geographic structure within the global SARS-CoV-2 phylogenetic tree.

**A Daily-Updated Website To Explore SARS-CoV-2 Clusters in the USA**

To make the results of this work broadly useful for the research and public health community, we have developed a visualization and exploration platform. Cluster-Tracker is a publicly-available, daily-updated website displaying the latest results for applying our heuristic to sequences collected from across the United States of America interactively (clustertracker.gi.ucsc.edu; see Methods; Figure 5). Cluster-Tracker is open-source with a flexible backend pipeline that allows any user to construct a similar site for any set of regions they have geographic information and sample identification for (https://github.com/jmcbroome/introduction-website).

Cluster Tracker is composed of two primary sections and some descriptive text (Figure 5). The first section is an interactive map of the United States. In the default view, this map is colored by the number of clusters detected across each state throughout the course of the pandemic. The true number of introductions into a given region is likely to be substantially larger because many small clusters will not be sampled by ongoing viral surveillance efforts, but major local transmission clusters should be represented. By clicking on a state, the site changes to a view specific to that state. In the default view, the map is colored by the log-fold enrichment of introductions from each other state to that state. Optionally, the user can switch the color to raw counts of detections with the toggle in the upper right.

The second section is a sortable, searchable table display of the highest priority clusters. In the default view, these are the top 100 clusters overall as sorted by "growth score". We define "growth score" as the square root of the number of samples divided by the number of weeks since the introduction occurred. The goal of this metric is to weight clusters by relative size and how recently they entered a given area, so that clusters of interest to public health appear first. When a state is selected, this table changes to the top 100 clusters obtained from that particular state. Basic information including clade, lineage, the earliest and latest dates of detection, and inferred origins are displayed for each cluster. The "inferred origin confidences" column is the highest or tied for highest regional index among all other regions for the parent node to the cluster origin, with a floor of 0.05 below which the cluster is simply marked "indeterminate". The "inferred origins" column is the regions which match these scores, and generally represents our best guess at the origin of this cluster. The last column of the table contains links to the Taxonium viewer (https://github.com/theosanderson/taxonium) which will automatically render the full tree and zoom to the cluster of interest when opened (Figure 6). Full results and the taxonium protocol buffer file, which encodes the tree and all cluster IDs, are available to be downloaded at the bottom of the page.

The goal of this resource is to make cluster identification, exploration, and prioritization more accessible and digestible for public health offices and policy makers. A significant roadblock for public health action is the sheer quantity of daily new data and the speed with which we can draw inferences from these data. Cluster-Tracker can assist exploration and prioritization of the latest genome sequences, quickly identifying the clusters most likely to be of interest for public health action for a given region. Our construction pipeline is flexible and can be applied for any set of regions (*e.g.,* county-level), allowing groups anywhere to construct web interfaces for intuitive SARS-CoV-2 phylogenetic data exploration.

While simple and efficient, our heuristic does exhibit some weaknesses. It is not a model; while simulations have demonstrated its efficacy in describing simple patterns of transmission, it can fail to correctly infer more complex scenarios and requires substantial and dense input data. Simulations indicate that it performs best with larger and more homogenous regions with low rates of migration, such as countries. If the user attempts to infer introductions with very small regions with high rates of inter-regional transmission, it may fail to properly recapitulate transmission patterns. Additionally, regionally biased differences in sequencing effort (Brito et al 2021, Colson et al 2021) can lead to significant biases in raw counts and our ability to correctly identify introductions, making individual cluster origins difficult to interpret in many cases. In terms of functional limitations, the heuristic is based on a binary regional labeling model, and does not have the ability to directly interpret lat-long coordinates or unique location values for samples like some Bayesian phylogeographic methods. Overall, it remains a useful tool for quickly assaying viral diversity and inter-regional transmission patterns on a global scale.

**Conclusion**

The pandemic has made the need for rapid and powerful tools to unlock the potential of pandemic-scale genomic epidemiology. The method we developed and the efficient software package we provide will empower researchers worldwide to make fast inferences from vast sequence datasets. Our results have revealed geographic structure at scales below the level of pango-lineage (O'Toole et al 2021) within the global SARS-CoV-2 phylogeny. We have provided tools and resources with which to explore this geographic structure and draw useful inferences for specific areas. Additionally, to empower public health officers and the public to explore the spread of SARS-CoV-2 across the USA, we developed an accessible open-source interactive interface for our results, which can automatically compute and display introductions and clusters with each update to the global phylogenetic tree. Our work can support public health groups across the world to quickly understand and apply insights obtained from the latest genomic data.

## Methods

### matUtils Implementation

We implemented a calculation of this heuristic as a part of our online phylogenetics package, matUtils, under the command "matUtils introduce" (McBroome et al 2021) (https://github.com/yatisht/usher). Our implementation uses dynamic programming based on a post-order traversal to compute the regional index for each node in the tree in a single pass for each region. This is because the four parameters which define regional index- distance to the nearest descendent and total descendents for in-region and out-of-region- can be computed from these same metrics for each child of a node plus the branch length to each child. The total number of leaves descended from a query parent node is the sum of all leaves descended from each of their children, and the shortest distance traversed to a leaf is the minimum of each child's minimum distance traversed plus the branch length between that child and the query parent. Therefore, by computing it first for nodes with only leaf children, then progressively deeper internal nodes, we only have to reference the children of each internal node and check their stored values instead of having to traverse from each node. This step is optionally parallelized across distinct regions, if multiple regions are passed.

The secondary step is an ancestry traversal for each sample in the tree, identifying the most recent ancestor which has a regional index below the set threshold, which is inferred to be the introduction point for this lineage. Once introduction points have been inferred for each sample, samples are grouped by shared introduction points into clusters, basic statistics and information are computed, and results are reported.

Ultimately, our implementation can compute this heuristic, identify clusters, and report all results in less than two minutes for a tree containing more than two and a half million samples (Supplementary Table 2). The speed of calculation is a major attraction of this heuristic approach over more complex Bayesian models. Calculating in minutes on minimal computing resources makes this method accessible and applicable to update results daily, identifying clusters and introductions as they occur and new data is uploaded globally. Accordingly, this implementation underlies our website Cluster-Tracker, which is updated with all new uploaded data each day and a recalculation of our heuristic.

### Handling Nested Clusters and Unstructured Regions

We implemented a few additional parameters that can be used to control behavior at the secondary cluster identification step. Once that is useful is setting a short-range maximum index requirement- that is, looking ahead at some additional number of ancestors and ensuring that each of those have a lower regional index than the intended ancestor node. Setting this parameter causes small nested clusters to be merged into larger overarching clusters. Another useful parameter is a minimum required branch length between the ancestor inferred to be in-region to its parent; if the branch length is less than the minimum, then the parent instead of the in-region node is inferred as the introduction point. Setting this parameter allows sibling clusters to be merged if both of their branch lengths are below minimum; this also resolves unstructured parts of the tree where large polytomies of identical samples with branch length 0 both in and out of a region are included.

**Prioritization and Bias Handling**

Another significant point of consideration is cluster prioritization. This cluster identification method is based solely on the phylogenetic tree and simple sample-region association, and while this makes it lightweight and flexible, identifying clusters which died out locally months ago is not of use to public health offices doing real-time transmission cluster tracking. We therefore in our implementation sort the output by a "growth score", defined as the square root of the number of samples associated with the cluster divided by the time in weeks from the oldest sample in the cluster to the current date plus one. This means that large, recent clusters will appear at the top of any output tables, and makes the method more easily accessible when thousands of clusters are being inferred simultaneously.

When using this method to examine inter-region transmission dynamics, we rely on comparable and significant levels of sequencing in order to identify introduction origins. Intuitively, the less sequencing is performed in a region, the less likely we are to recognize sequences from that region when they appear in another region. We can compensate for this bias to an extent by calculating log-fold enrichment of introductions between regions. This is computed as

$$Log\ Fold\ Enrichment\ (LFE)\ = log_{10}(\frac{Iab \times Ixx}{Iax \times Ixb})$$

Where $I_{ab}$ is introductions from region A to region B, $I_{xx}$ is introductions from any region to any region, $I_{ax}$ is introductions from region A to any other region, and $I_{xb}$ is introductions from anywhere to region B. This computation can remove biases in rates of detected introduction which would apply to any pair of regions, but requires many regions to be computed as points of comparison. This score is used to color the map on Cluster-Tracker when a state is selected and has a very strong correlation with geographic distance.

**Simulation for Validation**

To assay the performance of our heuristic, we fully simulated a pandemic phylogeny with VGsim (Shchur et al 2021) and phastSim (Maio et al 2021). From the resulting mutation-annotated tree, we calculated true node region states based on VGsim's migration event output and applied our heuristic with matUtils (McBroome et al 2021). We then computed accuracy as the proportion of internal nodes which have a heuristic value above 0.5 for the true state. Leaves are excluded from this calculation as they are taken as an input in our heuristic and will always be 100% accurate.

For our specific results, we simulated a one-million-leaf SARS-CoV-2 tree under a simple model with two equivalently-sized regions with an even rate of migration between them, no strain or site selection and complete immunity for recovered individuals (Supplementary Table 2). We included a lockdown parameter starting at 5% infected and ending at 1% infected, with a 10-fold reduction in transmissivity under lockdown, and a sampling multiplier of 0.2 in order to deepen the tree by effectively extending the time for one million samples to be collected.

ARI (Adjusted Rand Index) and IAC (Internal Assignments Correct) are our quality metrics. ARI represents how well our method correctly groups samples into true clusters descended from a single introduction event. ARI performs best when migration is low, leading to large and clean clusters which are easily separated heuristically, and performs somewhat better when scale is increased. IAC is the proportion of internal nodes which are assigned to the true region by our heuristic across the bifurcating tree. It is computed on the correct bifurcating tree because collapsing true nodes from different regions leads to nodes that are naturally indeterminate. IAC is generally robust, only performing slightly worse with an increased migration rate, likely as deeply set internal nodes tend towards indeterminacy with high distances to many leaves across different regions. This suggests that the primarily limitation of our heuristic is simply the number of mutations available to distinguish samples from across varying regions rather than any structural or fundamental issues.

All code for this simulation is available as a modular and reproducible Snakemake pipeline at github.com/jmcbroome/pandemic-simulator.

**Global Phylogenetic Tree Construction**

At UCSC we maintain a large phylogeny of all GISAID (Shu et al 2017), GenBank (Sayers et al 2021), and COG-UK (Lancet Microbe 2020) sequences using the script https://github.com/ucscGenomeBrowser/kent/blob/master/src/hg/utils/otto/sarscov2phylo/updatePublic.sh and the UShER online phylogenetics suite (McBroome et al 2021, Turakhia et al 2021). Updates are performed daily by obtaining all newly uploaded sequences from each database and placing them on the previous day's global phylogenetic tree with UShER (see McBroome et al). Starting with our phylogeny updated on 11-28-2021, we pruned all samples with long branch lengths and path lengths using the matUtils parameters *--max-branch-length 45* and *--max-path-length 100* and performed a round of optimization with an SPR radius of 8. The resulting phylogeny contained 5,563,847 samples with a total tree parsimony of 4,847,954.

**Computing USA state transmission**

We obtained the latest mutation-annotated phylogenetic tree representing the entirety of all public samples and all samples available on GISAID on 11-28-2021. As the standard format for publicly uploaded SARS-CoV-2 sequence identifiers is "Country/(Area)-CollectingAgencyInfo/Year|Date", we extracted sample labels for samples in the USA by identifying samples with names beginning with "USA/" and then extracting the two-letter state code, if it matches with a two-state letter code. This resulted in 1,764,019 labeled samples belonging to the USA. Samples from outside the USA were labeled by country; countries and ambiguous labels with less than 500 samples in GISAID and public data were excluded and their samples removed. Samples from "mink" were additionally excluded as they may not be from human sources. The resulting tree contained 5,237,796 of the total of 5,563,847 samples available, reflecting more than 94% of all SARS-CoV-2 genomic data collected and incorporated to date.

We applied matUtils introduce with default parameters to this tree and sample set and produced the full by-sample output. After computing basic statistics, we calculated log-fold enrichment of introductions between all pairs of states, and a selection of other countries to and from the USA. All code for this paper is provided at https://github.com/jmcbroome/cluster-heuristic).

**Cluster-Tracker Website Development**

All relevant javascript and some example data files are provided at (https://github.com/jmcbroome/introduction-website). This github includes a brief description of how to set up a local test site and run the backend pipeline for generating new results to display for your regions of interest. It is based on Leaflet (https://leafletjs.com/) and DataTables (https://datatables.net/) for the primary view, and includes links to the Taxonium tree viewer (https://taxonium.org/) for detailed cluster exploration.

We include Python scripts to create the backend data for the website display, contained in the “data” directory. This includes two versions of the primary pipeline. One is specific to the United States, which fills in many default parameters and uses data included in the repository. The second is a more flexible and configurable pipeline which given a tree, sample labels, and a geojson, can create a Cluster-Tracker equivalent website for any set of regions.

**Comparison with Published Studies**

To compare our approach to that of Alpert et al 2021, we retrieved the Auspice JSON they used to generate Figure 3 from (https://github.com/grubaughlab/CT-SARS-CoV-2) and obtained table S3 from their supplementary data online, which contains cluster labelings for samples from the tree represented by the JSON. We converted the Auspice JSON to the UShER MAT protocol buffer format using python. We labeled all samples in the resulting tree by their country of origin and ran matUtils introduce with default parameters. The resulting labels were compared to the cluster labels presented in table S3 and the Adjusted Rand Index was computed across all labeled samples with scikit-learn (Pedregosa et al 2011). We performed this analysis twice- once including all samples in their tree from any region and once excluding samples from the USA in their tree that were excluded from their clusters. The first method resulted in an ARI of 0.9 and the second a perfect 1.0; this discrepancy results from a single difference where a pair of large clusters, sibling to one another, are merged by our results when samples excluded from their clusters are included in our analysis. This is because a sample identical to the parent node of these two sibling clusters from the USA is excluded from Alpert et al’s clusters. In any case, the clusters we identify are highly concordant with Alpert et al’s results. All code for this analysis is available on (https://github.com/jmcbroome/cluster-heuristic).

**Acknowledgements**

We gratefully acknowledge helpful comments and feedback from Gage Moreno, as well as the submitting laboratories where genome data were generated and shared via GISAID (Shu et al 2017), COG-UK (Lancet Microbe 2020), and GenBank (Sayers et al 2021) and authors from the laboratories responsible for obtaining the specimens. Full sample credit is available in Supplementary Data 1.

**Funding**

JM was supported by T32HG008345. This work was funded in part by CDC award BAA 200-2021-11554 to RBC.

**Author Contributions**

JMB and RBC conceived and designed this research. JMB developed the heuristic, wrote the software, and performed comparison and validation experiments. JMB developed the website. JM contributed to the website and documentation for the website. JMB and RBC wrote the manuscript. JMB, RBC, and ABS edited the manuscript.

**Competing Interests**

The authors declare no competing interests.

**Replication and Data Availability**

Code to replicate our analysis is available at https://github.com/jmcbroome/cluster-heuristic.
Code for complete simulation of covid-like phylogenetic trees is available at https://github.com/jmcbroome/pandemic-simulator
Our implementation of our heuristic is implemented as part of matUtils https://github.com/yatisht/usher with additional documentation at https://usher-wiki.readthedocs.io/en/latest/
Our website source code is available at https://github.com/jmcbroome/introduction-website.
All data was obtained from the public repositories GISAID (Shu et al 2017), COG-UK (Lancet Microbe 2020), and GenBank (Sayers et al 2021), with full individual sample credits in Supplementary Data 1.

**Figure 1: Example Index Calculation.** This small example tree demonstrates a computation of our index, using blue to indicate “out-of-region” and red to indicate “in-region” leaves. The focal node at the base has an index value below 0.5, suggesting that it is out-of-region by our heuristic. Our introduction point is therefore along the long branch below the root, and the ancestor of the downstream in-region sample cluster would have existed along that branch.

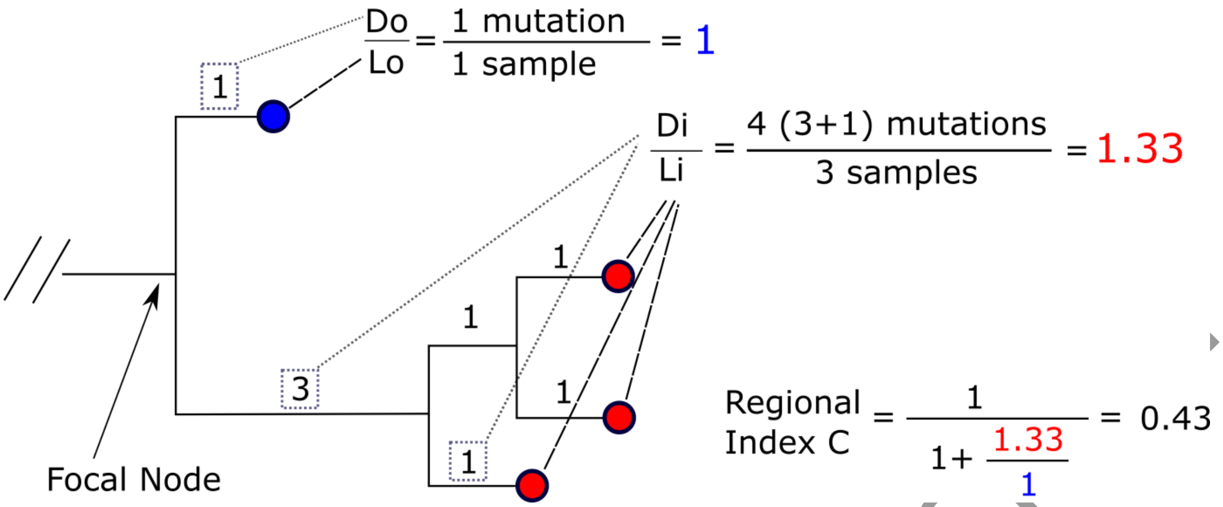

**Figure 2: Global Distribution of SARS-CoV-2 Transmission Clusters**. **A:** The log count of clusters detected across each of the 102 countries surveyed. The number of clusters detected is largely a function of total local sequencing effort. **B:** The five countries with the highest representation in the data. The USA and England together constitute more than half of all available sequences. **C:** Cluster sizes are consistent across countries. Most clusters are small, implying most newly introduced SARS-CoV-2 lineages quickly die out.

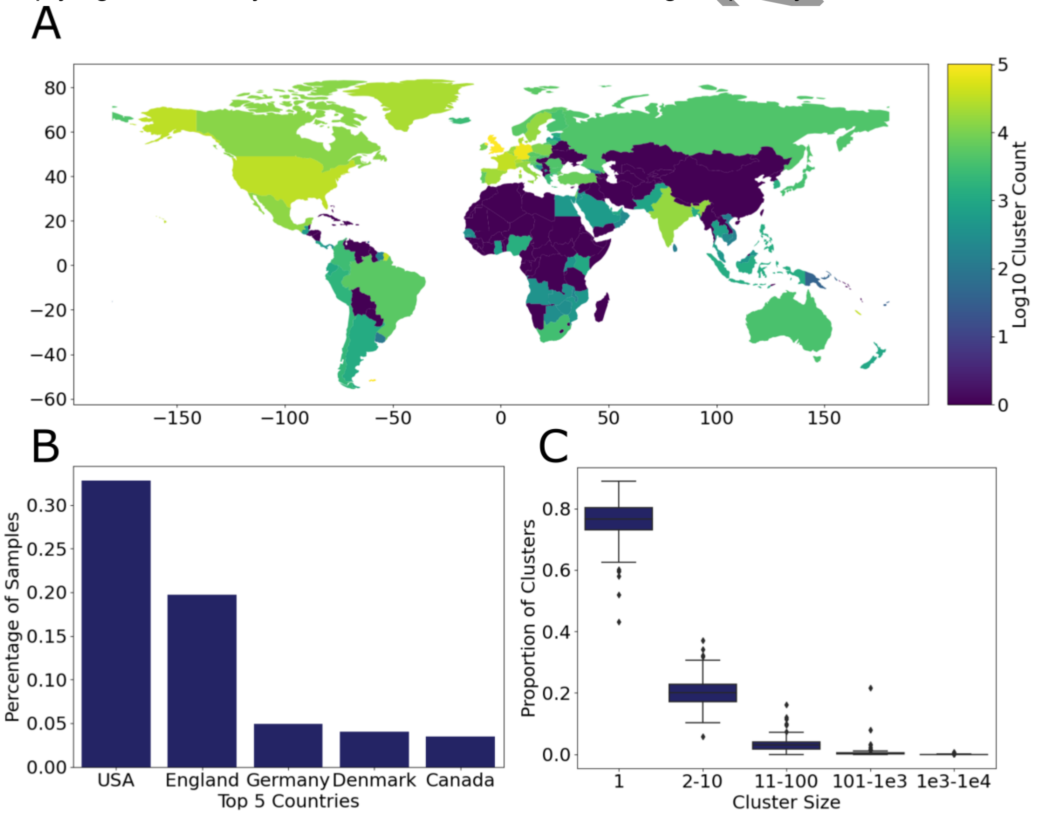

**Figure 3: International and Interstate Introductions across the USA. A:** The log count of clusters identified across the continental USA. California, Texas, Florida, and New York are associated with the greatest number of unique clusters. **B:** The proportion of international introductions in each state plotted against total samples collected in that state. This relationship is largely linear, reflecting the correlation between sampling, population size, and levels of international travel. PR (Puerto Rico) exhibits relatively more international introductions for its sampling than other territories and states of the United States. **C:** The distribution of cluster sizes across states. These are largely consistent with clusters identified at the international level.

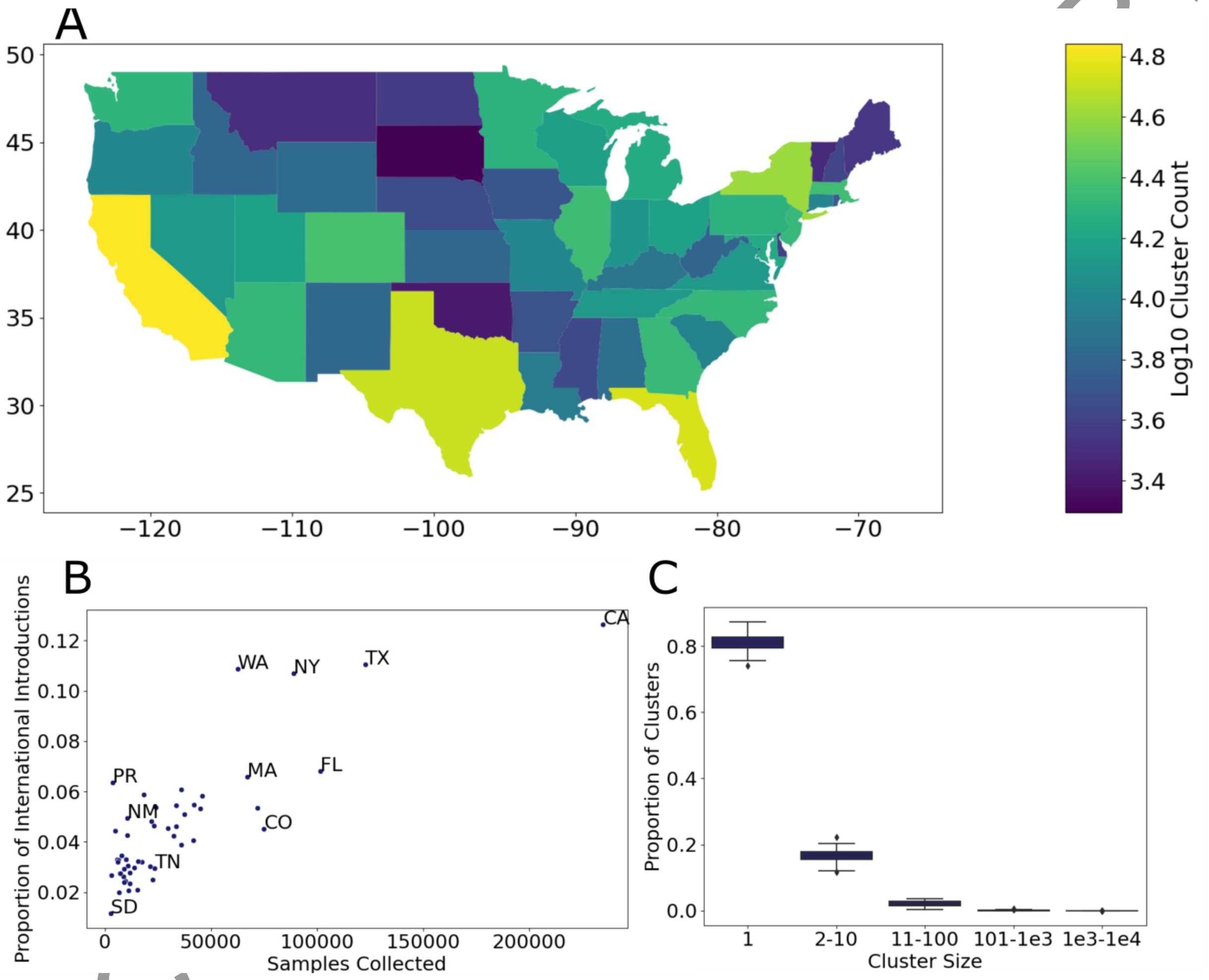

**Figure 4: Log-Fold Interstate Transmission for the States of California (A) and Illinois (B). A:** Interstate introductions of COVID-19 into California are relatively more likely to originate on the West Coast, particularly from Nevada. **B:** Interstate introductions of COVID-19 into Illinois are relatively more likely to come from the immediate surroundings, particularly Iowa and Missouri.

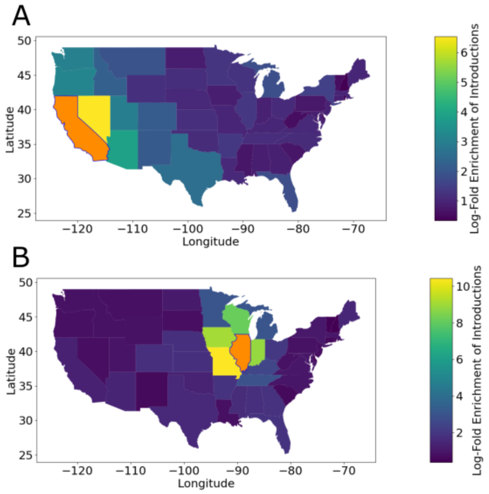

**Figure 5: the Cluster-Tracker site.** The Cluster-Tracker tool is updated daily at clustertracker.gi.ucsc.edu. Users can interactively explore the latest results of our heuristic applied to each of the continental United States, by sorting the interactive table, selecting states to focus on in the map, and using the Taxonium tree-viewing platform to examine clusters of interest in detail.

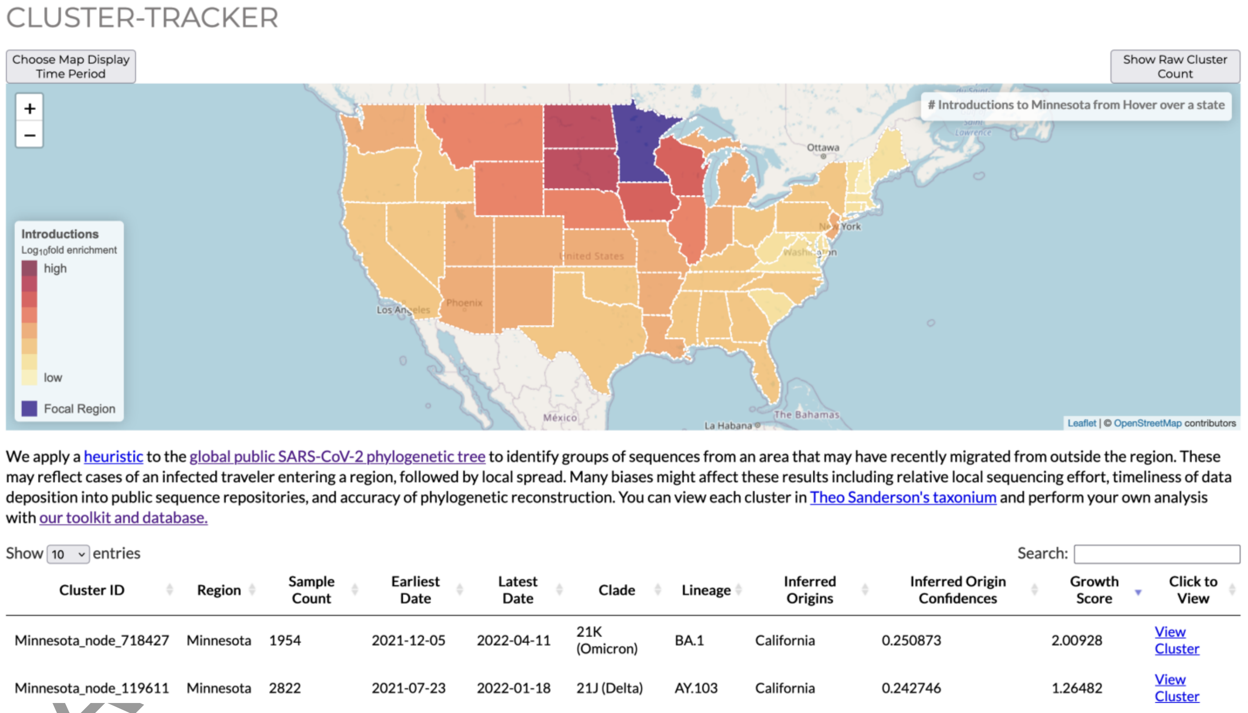

**Figure 6: Example clusters in the taxonium phylogenetic tree viewer.** (A) An example cluster in Texas (member samples circled in red) that is inferred to have originated from California (Regional Index = 0.94). There are many samples from California closely related to the cluster's common ancestor, supporting California as the most likely origin. (B) A different, much larger, 9,533 leaf cluster in California. This represents a lineage of SARS-CoV-2 commonly circulating in California, descended from one of the original introductions of the Delta variant into California in mid June 2021. Descendants from this cluster have transmitted to other

USCRIPT

regions many times, but members of this cluster have been found in California as recently as December 7th 2021.

## Supplementary Material

veac048_Supp

